# Epigenetic marks associated with gestational diabetes mellitus across two time points during pregnancy

**DOI:** 10.1186/s13148-023-01523-8

**Published:** 2023-07-06

**Authors:** Teresa Linares-Pineda, Nerea Peña-Montero, Nicolás Fragoso-Bargas, Carolina Gutiérrez-Repiso, Fuensanta Lima-Rubio, María Suarez-Arana, Antonio Sánchez-Pozo, Francisco J. Tinahones, María Molina-Vega, María José Picón-César, Christine Sommer, Sonsoles Morcillo

**Affiliations:** 1 Unidad de Gestión Clínica de Endocrinología y Nutrición, Instituto de Investigación Biomédica de Málaga-IBIMA_Plataforma Bionand, Hospital Universitario Virgen de la Victoria, 29010 Málaga, Spain; 2 Department of Endocrinology Morbid Obesity and Preventive Medicine, Oslo University Hospital, Oslo, Norway; 3 Faculty of Medicine, Institute of Clinical Medicine, University of Oslo, Oslo, Norway; 4 Centro de Investigación Biomédica en Red de Fisiopatología de la Obesidad y Nutrición (CIBERObn), Instituto de Salud Carlos III, 29029 Madrid, Spain; 5 Departamento de Obstetricia y Ginecología, Instituto de Investigación Biomédica de Málaga-IBIMA_Plataforma Bionand, Hospital Regional Universitario de Málaga, 29009 Málaga, Spain; 6 Departamento de Bioquímica y Biología Molecular 2, Universidad de Granada, Granada, Spain; 7 Departamento de Medicina y Dermatología, Universidad de Málaga, 29010 Málaga, Spain

**Keywords:** Gestational diabetes mellitus, DNA methylation, Epigenetics, Epigenome-wide association study, Diabetes, Pregnancy, Validation

## Abstract

An adverse intrauterine or periconceptional environment, such as hyperglycemia during pregnancy, can affect the DNA methylation pattern both in mothers and their offspring. In this study, we explored the epigenetic profile in maternal peripheral blood samples through pregnancy to find potential epigenetic biomarkers for gestational diabetes mellitus (GDM), as well as candidate genes involved in GDM development. We performed an epigenome-wide association study in maternal peripheral blood samples in 32 pregnant women (16 with GDM and 16 non-GDM) at pregnancy week 24–28 and 36–38. Biochemical, anthropometric, and obstetrical variables were collected from all the participants. The main results were validated in an independent cohort with different ethnic origin (European = 307; South Asians = 165). Two hundred and seventy-two CpGs sites remained significantly different between GDM and non-GDM pregnant women across two time points during pregnancy. The significant CpG sites were related to pathways associated with type I diabetes mellitus, insulin resistance and secretion. Cg01459453 (SELP gene) was the most differentiated in the GDM group versus non-GDM (73.6 vs. 60.9, *p *= 1.06E−11; FDR = 7.87E−06). Three CpG sites (cg01459453, cg15329406, and cg04095097) were able to discriminate between GDM cases and controls (AUC = 1; *p *= 1.26E−09). Three differentially methylated positions (DMPs) were replicated in an independent cohort. To conclude, epigenetic marks during pregnancy differed between GDM cases and controls suggesting a role for these genes in GDM development. Three CpGs were able to discriminate GDM and non-GDM groups with high specificity and sensitivity, which may be biomarker candidates for diagnosis or prediction of GDM.

**Supplementary Information:**

The online version contains supplementary material available at 10.1186/s13148-023-01523-8.

## Introduction

GDM is defined as a hyperglycemia with onset during pregnancy. The prevalence of this pathology varies between 1.8% and 31.5%, depending on the diagnostic criteria used and the population studied [1]. For women, GDM may increase the risk of prematurity, C-section delivery, preeclampsia, long-term risk of type 2 diabetes mellitus (T2DM), metabolic syndrome, renal disease, and cardiovascular disease (CVD). Women with a previous history of GDM have up to 10 times higher risk of developing T2DM, and half of them will develop some level of impaired glucose metabolism in the next 10 years after having GDM [2, 3]. The offspring of women with GDM have increased risk of fetal macrosomia, higher infant fat mass, and obesity [4]. Therefore, pregnancy is a unique window of opportunity to identify women and children at increased long-term risk of T2DM, obesity, and other cardiometabolic pathologies [5].

Although the severely increased long-term risk of T2DM after GDM is well-documented, the underlying molecular mechanisms are unclear. In last decades, epigenetic has been proposed as a potential link between genetic and environmental risk factors for GDM and T2DM [6]. Epigenetics refer to changes to the DNA that alter gene expression without altering the DNA sequence. These changes include DNA methylation, histone modification and, recently, non-coding RNA such as micro-RNA (miRNAs) [7].

Currently, most epigenetic studies in GDM have been carried out in offspring exposed to GDM [8–10]. However, few studies have explored the effect of GDM on the epigenome of the mothers and potential genes involved in the development of diabetes in pregnancy [11–13]. Further, none have so far tested the association with GDM across time points during pregnancy. Early identification of pregnant women with high risk of GDM and knowledge about the mechanisms responsible for development of GDM would help to improve diagnosis and treatment and to stop the vicious cycle of obesity and T2DM [14].

In this study, we explored the epigenetic profile in maternal peripheral blood samples through pregnancy to find potential epigenetic biomarkers for GDM, as well as candidate genes involved in GDM development.

## Results

### Characteristics of the discovery cohort (EPI-DG)

The main characteristics of the studied population are shown in Table 1. GDM and non-GDM pregnant women did not differ in age, gestational age, pre-pregnant weight and BMI, biochemical variables such as cholesterol, HDL-Chol, triglycerides, and homeostatic model of insulin resistance (HOMA-IR) (Table 1). However, the GDM group had a lower weight gain during the last trimester of pregnancy compared with the control group. This observation is probably due to the good metabolic control and adherence to the lifestyle recommendations of the GDM pregnant women after diagnosis. After dietetic recommendations were implemented, 37.5% of the GDM cases required additional pharmacological treatment (insulin).

**Table 1:** Characteristics of the study subjects

Comparison	Discovery cohort (EPI-DG)						Validation cohort (EPIPREG)								
	Basal (24/28 weeks) *n *= 32			Antenatal (36–38 weeks) *n *= 32			Complete cohort *n *= 472			European *n *= 307			South Asian *n *= 165		
	Non-GDM (*n *= 16)	GDM (*n *= 16)	*P*	Non-GDM (*n *= 16)	GDM (*n *= 16)	*P*	Non-GDM (*n *= 331)	GDM (*n *= 141)	*P*	Non-GDM (*n *= 234)	GDM (*n *= 73)	*P*	Non-GDM (*n *= 97)	GDM (*n *= 68)	*P*
Age	34.2 ± 4.5	33.8 ± 4.1	NS				29.4 ± 4.6	29.4 ± 4.8	NS	30.1 ± 4.5	30.14 ± 4.9	NS	27.9 ± 4.5	28.7 ± 4.7	NS
Gestational age (weeks)	27.6 ± 2.1	28.1 ± 2.8	NS	35.8 ± 1.4	36.25 ± 1.1	NS	27.9 ± 12.9	27.9 ± 13.0	NS	28.1 ± 12.5	28.0 ± 13.8	NS	27.5 ± 10.2	27.8 ± 12.1	NS
O’sullivan (mg/dl)	160.7 ± 16.3	172.9 ± 22.2	NS			NS									
Glucose 0’ (mmol/L)	4.5 ± 0.4	4.9 ± 0.6	0.05	4.3 ± 0.8	4.4 ± 0.9	NS									
Glucose 60’ (mmol/L)	8.2 ± 1.4	11.1 ± 1.2	< 0.001												
Glucose120’ (mmol/L)	6.9 ± 1.4	9.9 ± 1.2	< 0.001												
Glucose 180’ (mmol/L)	5.8 ± 1.1	8.3 ± 1.3	< 0.001												
Weight (Kg)	75.4 ± 11.3	76.0 ± 13.5	NS	78.7 ± 11.9	76.5 ± 13.1	NS	73.4 ± 13.2	77.4 ± 14.9	**2.0E**−**03**	76.5 ± 13.2	81.7 ± 15.3	**2.0E**−**03**	65.94 ± 9.9	72.8 ± 13.2	**1.0E−04**
Previous BMI	25.5 ± 4.1	25.8 ± 4.5	NS				23.8 ± 4.32	25.6 ± 5.37	**4.4E−04**	24.1 ± 5.01	26.3 ± 6.05	**5.00E−3**	23.0 ± 3.2	24.84 ± 4.4	**6.4E−03**
SBP (mm Hg)	104.2 ± 9.9	111.4 ± 15.4	NS	106.9 ± 12.8	109.1 ± 12.8	NS	103.9 ± 9.6	107.1 ± 9.2	**1.0E−03**	105.8 ± 9.5	110.1 ± 9.2	**1.0E−2**	99.2 ± 8.5	103.9 ± 8.2	**1.0E−03**
DBP (mm Hg)	69.1 ± 8.2	70.1 ± 7.8	NS	71.2 ± 6.5	73.6 ± 8.8	NS	67.1 ± 7.3	68.4 ± 6.7	**3.1E−03**	67.8 ± 6.9	69.7 ± 7.2	**2.4E−2**	65.48 ± 7.68	67.1 ± 5.8	NS
Cholesterol (mmol/L)	7.04 ± 1.2	6.7 ± 1.3	NS	7.1 ± 1.31	6.6 ± 1.4	NS	6.4 ± 1.04	5.9 ± 1.1	**1.3E−06**	6.5 ± 1.1	6.01 ± 1.1	**7.0E−4**	6.2 ± 1.02	5.7 ± 1.0	**8.4E−03**
HDL-chol (mmol/L)	2.1 ± 0.47	2.01 ± 0.4	NS	1.9 ± 0.3	1.9 ± 0.4	NS	1.9 ± 0.4	1.8 ± 0.4	**6.8E−04**	2.00 ± 0.4	1.8 ± 0.4	**1.1E−02**	1.9 ± 0.4	1.8 ± 0.6	**4.6E−02**
Tg (mmol/L)	2.2 ± 0.6	2.2 ± 0.5	NS	2.9 ± 0.9	2.8 ± 0.7	NS	1.9 ± 0.7	2.0 ± 0.7	NS	1.9 ± 0.7	1.9 ± 0.7	NS	1.9 ± 0.6	2.1 ± 0.7	NS
HbA1C (%)	5.1 ± 0.28	5.3 ± 0.37	NS	5.3 ± 0.3	5.8 ± 1.6	NS	5.1 ± 0.5	5.3 ± 0.3	**6.7E−11**	5.0 ± 0.3	5.2 ± 0.3	**4.3E−05**	5.2 ± 0.3	5.4 ± 0.3	**5.3E−05**
HOMA-IR	1.6 ± 0.7	2.1 ± 1.2	NS	3.5 ± 7.3	3.7 ± 4.2	NS	1.6 ± 0.6	2.1 ± 0.4	**1.0E−08**	1.5 ± 1.1	1.9 ± 0.9	**1.4E−02**	1.7 ± 0.7	2.3 ± 0.5	**6.0E−06**
Weight gain (Kg)				3.3 ± 1.4	0.49 ± 2.6	**1.0E−03**									

Data are expressed as the mean ± standard deviation. Proportions were compared by chi-square test and quantitative variables were analyzed using unpaired *t* test or Mann–Whitney U test according to normal distribution

Weight gain was calculated as the difference between the weight at the antenatal visit (T1) and at diagnostic visit (T0). *NS* non-significant

*BMI* Body mass index, *SBP* Systolic blood pressure, *DBP* Diastolic blood pressure, *HDL* High-density lipoprotein cholesterol, *Tg* Triglycerides

### DNA methylation pattern in pregnant women with GDM and non-GDM

A total of 1141 CpGs and 465 CpGs sites (FDR < 0.05, deltaBeta > 5% and *B* ≥ 0) were differentially methylated at diagnostic (*T*_0_) and antenatal visits (*T*_1_), respectively. The majority (66%) of DMPs were hypermethylated at both visits (*T*_0_ = 757, *T*_1_ = 311) in GDM compared to non-GDM group. Based on genome position, most of these DMPs were located in Open Sea, and according to gene context, mainly in body and IGR regions (Additional file 1: Fig. S1). Additionally, we evaluated if the epigenetic profile changed during pregnancy in both groups. We observed a total of 267 DMPs in the control group throughout time, whereas in the GDM group, we did not find any CpGs site differentially methylated over time (Additional file 2: Table S1). Lastly, we observed only two CpGs which responded differentially over time in the GDM group relative to the non-GDM group (Additional file 3: Fig. S2).

**Fig. 1:**
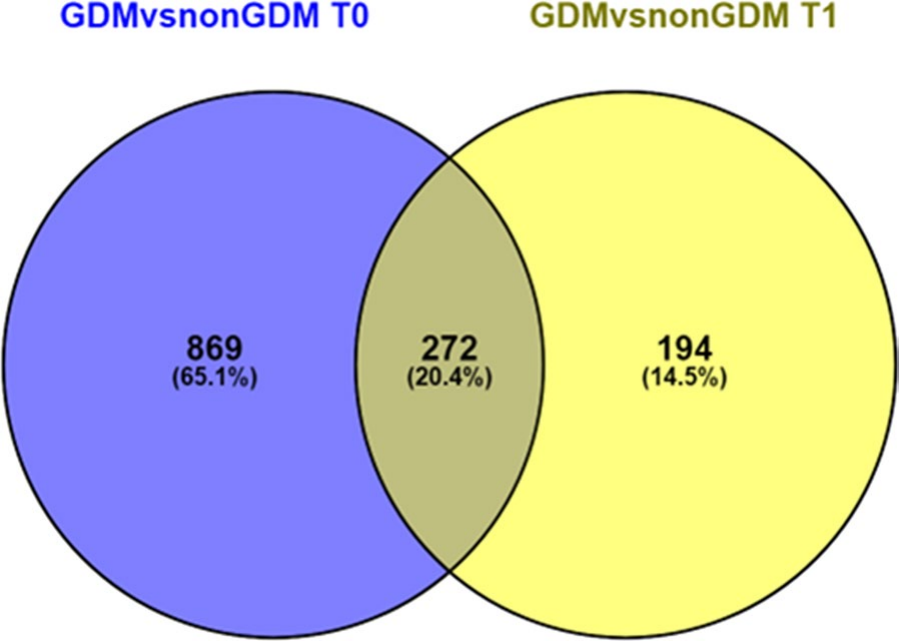
Venn diagram illustrating comparison of CpGs differentially methylated between pregnant women with GDM and non-GDM at baseline (blue) and antenatal visits (yellow). The CpGs that were maintained differentially methylated between both groups in both visits are shown in the middle

Using a Venn diagram, we observed that 272 CpGs sites were differentially methylated between GDM cases and controls across the two different time points (Fig. 1). From these CpGs sites, we selected the 20 most significant for further analysis. The top 20 DMPs were related to 12 genes (Table 2). Cg01459453, annotated to Selectin-P (*SELP*) gene was the most differentially methylated between both groups (12.7%), being hypermethylated in the GDM group. *NBL1* gene was specially enriched with three CpGs sites (cg18923740, cg15589641, and cg14579430) differentially methylated along the genome (body, TSS1500 and 5’UTR). No differentially methylated regions (DMRs) were found using the algorithm of DMRcate Bioconductor Package.

**Table 2:** Top 20 differentially methylated sites (DMPs) between pregnant women with GDM and non-GDM along the pregnancy

ID_CpG	LogFC	*P* value	FDR	Chr	Gene	Feature	Cgi
cg01459453	0.181	1.06E**−**11	7.87E**−**06	1	SELP	Body	Opensea
cg12432693	**−**0.057	4.07E**−**11	1.51E**−**05	11	OTOG	Body	Opensea
cg18217622	**−**0.206	8.73E**−**10	0.00016171	6		IGR	Island
cg18923740	0.090	2.18E**−**09	0.00032293	1	NBL1	Body	Island
cg12215871	0.115	2.89E**−**09	0.00035673	7	SDK1	Body	Opensea
cg04802986	0.118	6.75E**−**09	0.00071486	1	LGR6	Body	Opensea
cg21809624	**−**0.080	1.40E**−**08	0.00098142	17		IGR	Opensea
cg08386137	0.097	1.69E**−**08	0.00104499	6		IGR	Island
cg06279296	0.157	2.03E**−**08	0.00109495	10	DIP2C	Body	Opensea
cg27603605	0.077	2.61E**−**08	0.00129099	12	TMEM132B	Body	Opensea
cg15589641	0.112	3.35E**−**08	0.00145799	1	NBL1	TSS1500	Shore
cg10102108	**−**0.093	3.73E**−**08	0.00153659	20		IGR	Opensea
cg04600077	**−**0.082	5.04E**−**08	0.00169965	5		IGR	Shore
cg14688342	**−**0.073	5.53E**−**08	0.00169965	7	EGFR	Body	Opensea
cg07257824	**−**0.180	5.62E**−**08	0.00169965	6		IGR	Island
cg12080079	**−**0.132	6.27E**−**08	0.00177299	1	PAX7	Body	Opensea
cg23743013	0.078	7.00E**−**08	0.00177949	3		IGR	Opensea
cg14579430	0.120	7.09E**−**08	0.00177949	1	NBL1	5'UTR	Shore
cg01743873	**−**0.081	8.85E**−**08	0.0019774	11	CD151	5'UTR	Shore
cg01757548	0.084	9.00E**−**08	0.0019774	6		IGR	Island

*LogFC* log_2_ Fold Change, *Mean Controls* Mean of the *β*-value in control group, *Mean GDM* Mean of the *β*-value, *Chr* Chromosome location, *Feature* CpG location according with gene regions, *Cgi* CpG locations according with CpG islands, *IGR* Inter-genic region

### Gene ontology analysis

**Fig. 2:**
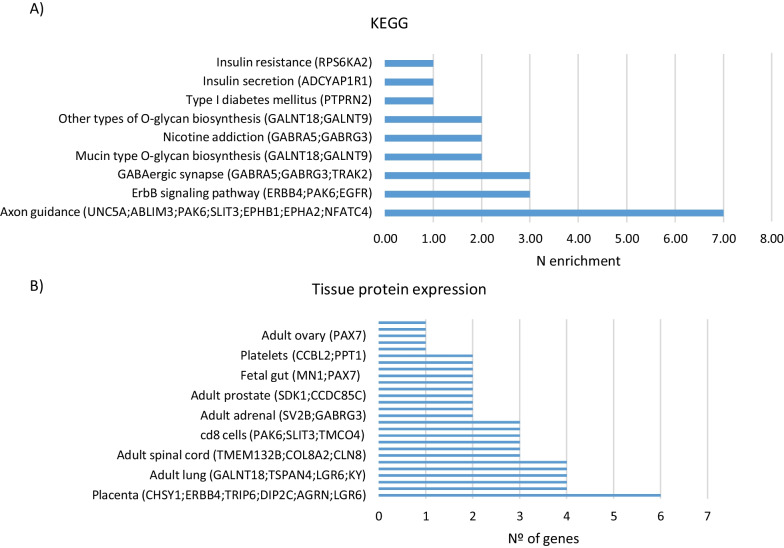
**A** KEGG pathways most enrichment sorted by inverse *p* value ranking. **B** Number of genes per tissue protein expression. Sorted by inverse *p* value ranking

Gene set enrichment analysis was performed using EnrichR and GO [15–17]. The 272 CpGs sites were annotated to 140 genes. Of them, 10% contained more than one probe differentially methylated (Additional file 4: Fig. S3). KEEG analysis identified six significant pathways related to Axon Guidance, ErbB signaling pathway, and calcium signaling pathway, among others. Moreover, relevant pathways related to type I diabetes mellitus (*PTPRN2*), insulin resistance (*RPS6KA2*), and insulin secretion (*ADCYAP1R1)* were also identified (Fig. 2). Analysis based on the Tissue Protein Expression database revealed that a group of genes annotated to our DMPs, such as *CHSY1, ERBB4, TRIP6, DIP2C, AGRN* and *LGR6*, showed higher expression levels in placenta than in other tissues (Fig. 2).

### ROC curve analysis of DMPs

**Fig. 3:**
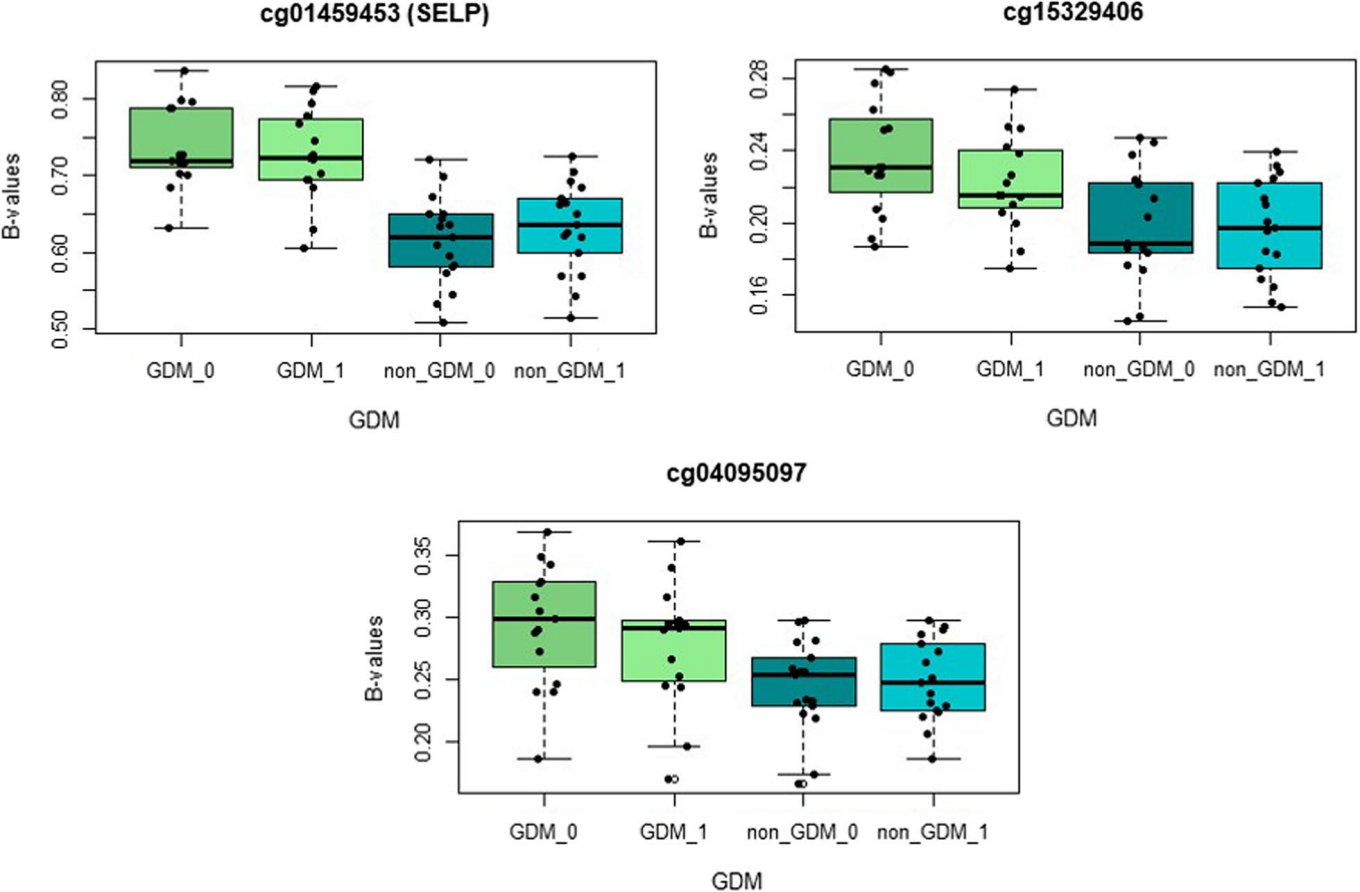
Box plots of the three CpGs statistically significantly included in the logistic regression model. Legend: GDM_0: Gestational diabetes mellitus group at T0. GDM_1: Gestational diabetes mellitus group at T1. Non_GDM_0: Non-gestational diabetes mellitus group at T0, Non_GDM_1: Non-gestational diabetes mellitus group at T1

We selected the 272 CpGs and performed ROC curve analysis to identify whether these could differentiate GDM cases from non-GDM. From the 272 CpG sites, 104 were statistically able to discriminate between GDM cases and controls (AUC ≥ 0.8) at diagnostic visit. The same approach was performed with the following clinical variables: HOMA-IR, age, gestational age, pre-pregnant BMI, and the glucose values at each point of the OGTT. Between the clinical variables, only the glucose values of OGTT had an AUC statistically significant ≥ 0.8 (AUC_SOG_60_ = 0.047; AUC_SOG_120_ = 0.025; and AUC_SOG_180_ = 0.043). We performed a logistic regression analysis to identify the variables that best discriminated between GDM cases and non-GDM, including all CpGs sites and glucose values from OGTT that had significant ROC curves. Of all the variables, three CpGs (cg01459453, cg15329406, and cg04095097) stood out as the most significant discriminators (Table 3). ROC figures are shown in Additional file 5: Fig. S4. Pregnant women with GDM showed a significantly higher DNA methylation in cg01459453 (*SELP*) compared with non-GDM women (73.6% vs. 60.9%, *p *= 1.10^–07^). The same significant trend was observed for cg15329406 and cg04095097 (Fig. 3).

**Table 3:** ROC analysis to evaluate the best discriminators of GDM versus non-GDM

Model	Sensitivity	Specificity	Precision	AUC	*P* value
Model 1 cg01459453	93.8	93.8	93.75	0.969	3.45E**−**08
Model 2 cg01459453, cg15329406	100	100	100	1	2.32E**−**10
Model 3 cg01459453, cg15329406, cg04095097	100	100	100	1	1.26E**−**09

*AUC* Area under the curve

### Validation in EPIPREG cohort

**Fig. 4:**
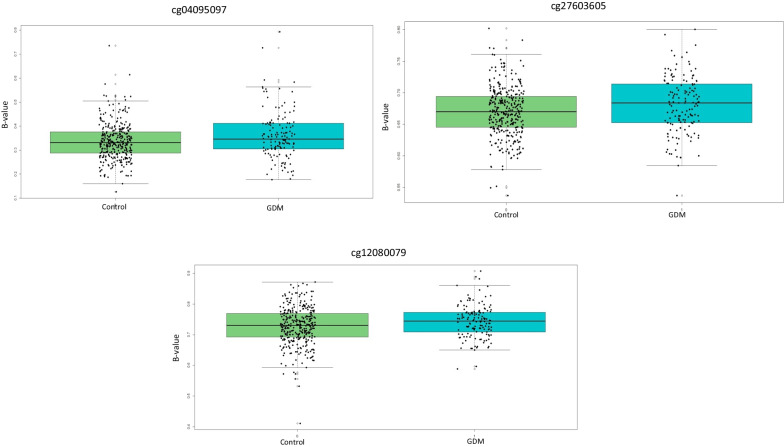
Box plot of the three CpGs validated in EPIPREG cohort. Legend: control: non-GDM; GDM: Gestational diabetes mellitus. All the three CpGs were statistically significant with *p* values < 0.05

The characteristics of EPIPREG are shown in Table 1. In EPIPREG, we attempted to replicate the top 20 DMPs and the three DMPs identified as the best discriminators of GDM. Cg04095097 was replicated in the full sample (*p *= 0.004), and this persisted after adjustment for covariates (Table 4). Of the top 20 DMPs, two CpGs were statistically different between GDM and non-GDM in the full EPIPREG sample (cg27603605 and cg12080079) (Table 4). Like the EPI-DG cohort, DNA methylation levels of these DMPs were higher in GDM pregnant women in comparison with non-GDM (Fig. 4). When stratifying by ethnicity, we observed statistically significant differences only for cg04095097 in the South Asians pregnant women (0354 non-GDM vs. 0.397 GDM; *p *= 0.026), and a trend in Europeans (Additional file 2: Table S2). Finally, a mixed models logistic regression showed that pregnant women with higher methylation levels of cg04095097 had a greater odd for GDM than women with lower methylation levels in the EPIPREG sample (OR = 1.25; 95% CI = 1.01–1.52).

**Table 4:** CpGs validated in EPIPREG cohort adjusted by age, pre-pregnant BMI, gestational age, HOMA-IR, fetal sex, and ancestry

	Log FC	Mean non-GDM	Mean GDM	*P* value
**cg04095097**	**0.16912107**	**0.3358**	**0.3613**	**0.00424159**
**cg27603605**	**0.07076299**	**0.669**	**0.681**	**0.01647897**
**cg12080079**	**0.09568801**	**0.728**	**0.743**	**0.04782572**
cg15329406	**−**0.0089716	0.29	0.288	0.80083925
cg01459453	**−**0.0147631	0.751795	0.7527223	0.81069312

*LogFC* log_2_ Fold Change, *Mean Controls* Mean of the *β*-value in control group, *Mean GDM* Mean of the *β*-value

Finally, when we performed ROC curve analysis with these CpGs in EPIPREG cohort, we obtained AUC statistically significant, although with an AUC lower than EPI-DG, for the following CpGs sites: cg04095097 (AUC = 0.570, *p *= 0.015), cg04095097 + cg27603605 (AUC = 0.597, *p *= 0.0009) and a combination with the three CpGs (AUC = 0.605, *p *= 0.0003) (Additional file 6: Fig. S5).

### mQTLs

From look-ups in GoDMC, we found 21 mQTLs for the three CpG sites replicated. According to Phenoscanner, the mQTL for cg12080079, rs585075, was associated with GDM (*p *= 0.03) (Table 5). Likewise, the other mQTLs were nominally related to T2DM, several cardiometabolic traits and outcomes, and inflammatory processes such as hypertension, psoriasis, Crohn’s disease, or rheumatoid arthritis (Table 5).

**Table 5:** mQTLs related to the three CpG sites replicated in EPIPREG

ID_cg	Gene	Rs	Gene	Position	A1	A2	Disease (PMID)	Beta	*P* value
cg12080079	PAX7	rs10907325	PAX7	chr1:18964021	A	T	Systolic blood pressure (PMID: 19609347)	NA	2.02E**−**05
							Body mass index within family tests max measurement (PMID: 21701565)	NA	2.50E**−**04
							Amyotrophic lateral sclerosis (PMID: 28931804)	**−**0.0112	7.33E**−**04
							Non-insulin-dependent diabetes mellitus (PMID: UKBB)	0.0002683	6.98E**−**03
		rs115326690		chr1:19036153	C	G	Systolic blood pressure (PMID: UKBB)	**−**0.02669	6.33E**−**04
							Hypertension (PMID: UKBB)	**−**0.01019	2.57E**−**03
							Psoriasis (PMID: UKBB)	**−**0.0007364	2.76E**−**03
		rs12563020		chr1:19022599	A	G	Type II diabetes adjusted for BMI (PMID: 28566273)	0.099	4.60E**−**03
							Type II diabetes (PMID: 28566273)	0.064	2.70E**−**02
		rs585075		chr1:19021373	T	C	Gestational diabetes only (PMID: UKBB)	**−**0.01402	2.56E**−**02
		rs7527312		chr1:19014865	C	G	Body mass index adjusted for smoking in males (PMID: 28443625)	0.0221	3.18E**−**03
cg04095097		rs10906900	FAM171A1	chr10:15472860	A	G	High-grade serous ovarian cancer (PMID: 28346442)	**−**0.05032	2.84E**−**03
		rs111256448	ITGA8	chr10:15765261	C	T	Crohn’s disease (PMID: 26192919)	**−**0.1337	1.27E**−**03
							Diastolic blood pressure (PMID: UKBB)	**−**0.01169	5.94E**−**03
		rs111920126	ITGA8	chr10:15510921	A	G	Clear cell ovarian cancer (PMID: 28346442)	**−**0.492	9.00E**−**04
		rs11259597	FAM171A1	chr10:15332911	A	G	Crohn’s disease (PMID: 26192919)	**−**0.211	3.73E**−**03
							Spondylosis (PMID: UKBB)	0.001257	4.26E**−**03
		rs11259690	ITGA8	chr10:15521768	A	G	Heart attack (PMID: UKBB)	**−**0.002578	1.36E**−**03
							Type 2 diabetes (PMID: UKBB)	**−**0.000909	3.31E**−**02
		rs114410649	ITGA8	chr10:15555993	A	G	Non-insulin-dependent diabetes mellitus (PMID: UKBB)	0.0006851	3.28E**−**02
		rs117164512	FAM171A1	chr10:15298845	C	T	Alcoholic hepatitis (PMID: UKBB)	**−**0.004734	3.62E**−**03
		rs12266863	ITGA8	chr10:15526023	A	G	Rheumatoid arthritis (PMID: 24390342)	**−**0.08338	8.20E**−**03
		rs12355715	ITGA8	chr10:15534600	C	T	Polyarthrosis (PMID: UKBB)	**−**0.000452	3.96E**−**03
		rs137882390	FAM171A1	chr10:15265022	A	G	Multiple valve disease (PMID: UKBB)	0.0006208	1.12E**−**03
							Illnesses of father: diabetes (PMID: UKBB)	**−**0.008409	6.48E**−**03
		rs187688289	FAM171A1	chr10:15471793	C	T	Low grade serous ovarian cancer (PMID: 28346442)	**−**0.6751	7.10E**−**04
		rs61514659	FAM171A1	chr10:15467588	C	T	Type 2 diabetes (PMID: UKBB)	**−**0.002412	6.07E**−**04
		rs7087034	ITGA8	chr10:15738818	C	T	–		
		rs71493225	ITGA8	chr10:15536804	C	T	Ischemic cardiomyopathy (PMID: UKBB)	0.002818	3.17E**−**04
							Insulin-dependent diabetes mellitus (PMID: UKBB)	0.000251	2.98E**−**02
		rs77070234	RP11-455B2.9	chr10:15226599	A	C	Eye problems or disorders: diabetes related eye disease (PMID: UKBB)	0.003402	8.50E**−**03
		rs7915524	FAM171A1	chr10:15293903	C	T	Self-reported mitral regurgitation or incompetence (PMID: UKBB)	0.0001378	2.50E**−**03
							Insulin-dependent diabetes mellitus (PMID: UKBB)	**−**0.000197	4.12E**−**02
cg27603605		Non-SNPs							

*UKBB* United Kingdom BioBank (www.ukbiobank.ac.uk), *A1* Allele dominant, *A2* Allele recessive

## Discussion

To the best of our knowledge, this is the first study analyzing the epigenetic profile in peripheral blood samples of pregnant women with and without GDM in different times during pregnancy. We identified 272 DMPs that were differently methylated between GDM and non-GDM across two time points during pregnancy, and several genes that could be involved in the pathophysiology of GDM such as *SELP*, *LGR6, NBL1, RPS6KA2* or *ADCYAP1R1.* We also detected three DMPs, cg01459453, cg15329406, and cg04095097, that adequately discriminated GDM occurrence and replicated one of these in an independent cohort.

Most epigenetic studies of gestational diabetes research have been carried out in placenta and cord blood samples [18]. Only a few studies have evaluated the epigenetic pattern in blood samples from pregnant women with GDM and non-GDM [19]. Although this approach cannot infer causality, the findings are very interesting as biomarkers and potential genes involved in the pathogenesis of GDM.

### Potential pathways and genes involved in GDM

We identified several CpGs annotated to genes related with relevant metabolic pathways. The CpGs most differentially methylated (cg01459453) between both groups of pregnant women was annotated to *SELP* gene. *SELP* gene encode for P-Selectin, a granular membrane protein and a cellular adhesion molecule that mediates the interaction of activated endothelial cells or platelets with leukocytes. Several epidemiological studies suggest that the endothelial dysfunction is closely related to insulin resistance, preceding the development of T2DM [20, 21]. This impaired function can decrease the delivery of insulin to the interstice, limiting the insulin action [20]. Likewise, other authors have found that high levels of P-selectin are associated with metabolic syndrome, and its expression and secretion have been related to low HDL cholesterol and elevated fasting glucose [22]. Another of the most significantly CpG associated with GDM in our study was cg04802986 located within the leucine-rich repeat-containing G-protein coupled receptor 6 (*LGR6*). CpG sites from this gene in the offspring’ epigenome were recently associated with maternal GDM, fasting glucose, 1-h, and 2-h glucose levels following an OGTT [23]. These epigenetic marks were attenuated by an intervention with diet and physical activity during pregnancy [23], suggesting that the effect of high maternal glucose levels on DNA methylation in the offspring could be modified by a lifestyle intervention.

*NBL1* and *DIP2C* had several DMPs differentially methylated*. NBL1* showed four CpGs sites differentially methylated, three of them within the top 20 DMPs. *NBL1* belongs to the DAN gene family, whose proteins are secreted and act as bone morphogenetic protein (BMP) antagonists avoiding the interaction with their receptors. Thus, they can play an important role during growth and development [24]. Just like *SELP,* several CpGs sites have been associated with age-related processes [25] as well as with gestational age [26]. Moreover, CpGs from NBL1 has also been associated with pre-pregnancy maternal BMI in a meta-analysis performed within the pregnancy and childhood epigenetics cohort (PACE) [27]. Regarding to *DIPC2*, this gene encodes a member of the disco-interacting protein homolog 2 family although its function is not fully understood. According to the literature, DIP2c gene is very susceptible to be modified by external factors [28, 29], so the hyperglycemia during pregnancy could be another modifier factor. Finally, our enrichment analysis identified within our DMPs, relevant pathways related to type I diabetes mellitus (*PTPRN2*) insulin resistance (*RPS6KA2*) and secretion (*ADCYAP1R1*). These results have been confirmed by several studies. For example, *RPS6KA2* has been related with insulin signaling pathways and T2DM complications [30, 31].

Few studies have analyzed the DNA methylation in maternal blood in women with and without GDM. In line with our findings, Wu et al. [11] evaluated changes in the epigenome from pregnant women prior to GDM development by genome-wide 450 K array. They identified five CpGs (*COPS8, PIK3R5, HAAO, C5orf34,* and *CCDC124* genes) as potential clinical biomarkers for early detection of GDM and therapeutic intervention. These authors validated these results by pyrosequencing, finding a good correlation with *β*-values, but they did not replicate the findings in other cohorts. Dias et al. [12] examined the relationship between GDM and DNA methylation profile in peripheral blood cells in South African women. They compared the epigenome by EPIC Illumina array in pregnant women with GDM and non-GDM at the first trimester. Just like the study by Wu et al., several CpGs sites were differentially methylated, and the associated genes were involved in pathways of metabolic regulation. Additionally, the top five CpGs were correlated with relevant metabolic variables such as glucose and insulin concentrations. Kang et al. [32] analyzed the epigenome in maternal and cord blood samples from 16 pregnant women (eight with GDM) and their offspring [33]. They found a differentially methylated pattern between GDM and unexposed group in both types of samples, supporting the hypothesis that the GDM has epigenetic effect on both mothers and their offspring.

### Potential epigenetic biomarkers of GDM

Due to the great controversial and variety about the diagnostic criteria for GDM, there is an increasing need to establish biomarkers able to identify in early pregnancy the occurrence of GDM. Currently, the Gold Standard is made with an OGTT at around 26–28 weeks of gestation but it is known that differences in fetal growth occur even at early pregnancy (at 12 weeks) between mothers who will be diagnosed with GDM and who will be not [34]. Finding out accessible and stable biomarkers for predicting GDM would allow an early intervention and the subsequent reduction risk both in mothers and their offspring. A recent study based on integration of EWAs and machine learning has identified 62 specific CpGs sites related to GDM, and six of them located in the promoter region, which were included in a predictive model for GDM whose AUC were for training and testing set, 0.8138 and 0.7576, respectively [35]. Just like us, CpGs sites within *DIP2C* and *PTPRN2* genes were differentially methylated between GDM and non-GDM pregnant women. Another group, led by Enchun et al. [36], has identified DNA methylation sites as potential biomarkers of GDM diagnostic, integrating high-throughput methylation and gene expression data . Although they identified ten genes related with GDM development, the study has several limitations, such as the absence of experimental validation and lacking confounding variables. Wang et al. [37] measured specific CpGs sites, previously published in literature, in 80 GDM cases and 80 matched controls in the first trimester to test if those target CpGs could be associated with GDM pathophysiology in order to be considered as potential predictor of the disease. Overall, a total of 13 CpGs sites showed DNA methylation levels statistically significant between GDM and non-GDM, and the AUC ranged from 0.593 to 0.650 to predict the risk of GDM. Some of the annotated genes, such as *NFATC4* or the family of *ARHGAP*, have been also associated in our study. Recently, a study carried out by Megía et al. [13] has identified several CpGs sites as possible biomarker to detect risk for development glucose abnormalities later in life in women with GDM . The CpGs sites were associated with *LINC00917, TRAPPC9*, and *LEF1* genes.

We identified three CpGs sites in the EPI-DG cohort with high capacity to discriminate between GDM and non-GDM pregnant women, whereof cg04095097 was replicated in the EPIPREG sample. An important characteristic from our study is that we have found stable epigenetic marks during pregnancy able to differentiate GDM and non-GDM groups, suggesting that maybe these marks could be at early pregnancy. In EPI-DG, pregnant women with GDM and non-GDM from the discovery cohort were matched by age, gestational age, and pre-pregnant BMI, whereas the replication cohort was not matched by any variables. Hence, the replication cohort is more heterogeneous than the discovery cohort, as suggested by the statistically significant differences in several variables between women with and without GDM. Maybe this could explain why these DMPs had less capacity to discriminate GDM versus non-GDM in EPIPREG cohort. However, the replicated CpG sites seem robustly associated with GDM, as they were common in two independent cohorts using different GDM criteria and across ethnic origin. Epigenetic marks could help to find a universal diagnostic criterion for GDM. Additionally, mQTLs found suggest that these CpGs are influenced by genetic variants associated with GDM, cardiometabolic traits and autoimmune disease [38–40].

Our study has important strengths. Firstly, we have analyzed DNA methylation at two different points during pregnancy. In the discovery cohort, GDM cases and non-GDM were very homogeneous, reducing the likelihood of bias or confounding variables to drive our results. A limitation of our study is that the replication cohort used different GDM criteria and women with and without GDM were not matched. Hence, a replication cohort with a more similar design could have provided better replication results. As limitation, we could not analyze the effect of DNA methylation on gene expression due to lack of RNA samples. Finally, further studies with higher sample size and at early pregnancy are needed to confirm our results and the potential of these epigenetic marks as biomarkers of GDM occurrence.

## Conclusion

We identified differentially methylated marks between GDM and non-GDM pregnant women at two different time points during pregnancy. Several of these DMPs are within genes associated with metabolic pathways related to insulin and glucose metabolism, pointing out a possible role in the GDM development. Furthermore, we have identified three CpGs sites that may pose as potential biomarkers for diagnosis of GDM.

## Methods

### Subjects

Pregnant women who attended Unit of Diabetes and Pregnancy at University Hospital Virgen de la Victoria, after a positive O’Sullivan test, were eligible for recruitment. GDM was diagnosed using a two-step strategy according to National Diabetes Data Group NDDG criteria [41]. Firstly, a screening test in pregnant women between 24–29 weeks of pregnancy, with a 50 g oral glucose load, was done in primary centers. An oral glucose tolerance test (OGTT-100 g) was carried out in those women with a positive screening test (> 7.7 mmol/L). Patients were diagnosed with GDM if glucose values were higher than the threshold, at least in two points: fasting > 5.8 mmol/L; after 1 h > 10.6 mmol/L; after 2 h > 9.2 mmol/L; and after 3 h > 8.0 mmol/L. Those pregnant women with normal OGTT-100 (NGT) were considered as controls (non-GDM). After GDM diagnosis, women were recommended to make lifestyle changes and self-monitoring of blood glucose (SBGM) at fasting and 1-h postprandial breakfast, lunch and dinner (Bayer, Contour® Next Glucose test strips, XT or USB meters). Diet recommendations included 175 g of carbohydrate, a minimum of 71 g of protein, and 28 g of fiber, avoiding saturated fat and simple carbohydrates and preferring a moderate consumption of complex carbohydrates. These recommendations were maintained during the whole study. After 1 week, glycemic controls were analyzed by the endocrinologist. If ≥ 2 glucose fasting values were ≥ 95 mg/dl (5.3 mmol/L) and/or 1-h postprandial ≥ 140 mg/dl (7.8 mmol/L) despite lifestyle changes, the addition of pharmacological treatment (insulin) was indicated.

Blood samples were collected in two different time, at diagnostic visit *T*_0_ (24–29 weeks) and at antenatal visit *T*_1_ (36–38 weeks). This cohort is part of the EPI-DG study which started at the beginning of 2019. Characteristics of this cohort has been recently published [42].

An epigenome-wide DNA methylation analysis (EWAS) was performed in 16 pregnant women with GDM and 16 non-GDM matched by age, gestational age, and pre-pregnant BMI to avoid confounding factors in the methylation data analysis. Clinical, anthropometric, and biochemical variables were collected in each visit. Weight gain was calculated as the difference between the weight at the antenatal visit (*T*_1_) and at diagnostic visit (*T*_0_).

All patients gave their consent to participate in the study. The study was approved by the Institutional review board at the Hospital Universitario Virgen de la Victoria de Málaga, Spain.

### Samples extraction, DNA isolation and bisulfite conversion

Blood samples were collected in each visit (*T*_0_ and *T*_1_) after a 12-h fast and stored at -80ºC until DNA isolation. Peripheral blood DNA was isolated using Qiamp DNA Blood Mini Kit (Qiagen, Hilden, Germany) according to the manufacturer’s instructions. Quality and concentration of DNA was measured using Qubit 3.0 Fluorometer with Qubit dsDNA HS Assay Kit Fluorometer (Thermo Fisher Scientific, Waltham, MA, USA). A total of 500 ng of genomic DNA was bisulfite treated with Epitect Bisulfite Kit (Qiagen, Germany) for posterior DNA methylation analysis.

### Epigenome-wide DNA methylation analysis

An Epigenome-wide DNA methylation analysis (EWAS) was performed in a total of 32 pregnant women, 16 GDM and 16 non-GDM (discovery cohort). DNA was hybridized in the Infinitum MethylationEPIC Bead Chip and 850.000 CpGs sites were analyzed. Raw data files were processed using R package ChAMP version 2.9.10 [43], filtering probes is performed in probes with a detection *p* value above 0.01 in one or more samples, probes with a beadcount less than 3 in at least 5% of samples, probe non-CpG, probes with SNPs [44], probes that align to multiple locations [45], and probes on the X or Y.

Intra-cell type normalization was done using beta-mixture quantile normalization (BMIQ) method. To correct for the differences in methylation resulting from differences in cellular heterogeneity, the Houseman correction was used [46].

### Methylation data analysis

The differentially methylated positions (DMPs) were obtained using eBayes moderated t statistic with limma package [47] for R statistical software (4.0.4). *β*-values and M-values were calculated to obtain the methylation levels. While *β*-values is the estimate of methylation level using the ratio of the methylation probe intensity and the overall intensity, it is used for report results. M-value is a logarithmic transformation of *β*-value, and it is necessary to perform the differential methylation analysis. Linear models were used to identify differentially methylated CpGs sites (DMPs) between GDM and non-GDM samples (FDR-adjusted *p* value < 0.05, and deltabelta ≥|5|%.) at both times. A Venn diagram was used to select those DMPs common in both visits (Venny 2.1 https://bioinfogp.cnb.csic.es/tools/venny/). All models were adjusted by age, pre-pregnant BMI, newborn sex, weight gain during pregnancy, gestational age, HOMA-IR, and required treatment. These variables have been previously associated with GDM in the literature. Additionally, weight gain was also included due to this variable was statistically different between groups.

A gene ontology (GO) and enrichment analysis were performed with those CpGs that were differentially methylated both *T*_0_ and *T*_1_ visits. Gene ontology tool website [17] and EnrichrR [24] were used for GO and enrichment analysis, respectively. Tissue protein expression database was used to explore gene expression levels in different tissues [48].

### ROC analysis and Logistic regression

Receiver operating characteristics (ROC) curves were performed to determine the AUC of the differentially methylated CpGs between GDM and non-GDM pregnant women. Variables with AUC ≥ 0.8 and *p *< 0.05 were selected. To generate the predictive models, binomial logistic regression was used as the dependent variable GDM. The ROC (receiver operating characteristic) analysis has allowed evaluating the generated models using different metrics such as sensitivity, specificity, precision, and area under the curve (AUC). We used R software (4.0.4) for this statistical analysis.

### Replication

For replication, we used the EPIPREG sample, which is a sub-study of the population-based STORK Groruddalen (STORK G) cohort [49].

In EPIPREG, we quantified DNA methylation in maternal peripheral blood leukocytes in gestational week 28 ± 2 in all Europeans (*n *= 312) and South Asians (*n *= 168) participating in STORK G who were genotyped and had fasting glucose data recorded, with Infinium MethylationEPIC BeadChip (Illumina, San Diego, CA, USA). Three hundred and seven Europeans and 165 South Asians passed the quality control. Details about the EPIPREG sample have been described previously [50].

All women completed a 75 g oral glucose tolerance test in gestational week 28 ± 2. Fasting and 2-h glucose was analyzed with a point-of-care instrument (HemoCue, Angelholm, Sweden). We classified GDM in retrospect with a modified version of the WHO 2013 criteria (fasting glucose ≥ 5.1–6.9 mmol/l or 2-h glucose ≥ 8.5–11 mmol/l, no data for 1-h glucose) [51].

We performed an eBayes moderated t statistic with the limma package, adjusted for the same variables as in the main analysis of EPI-DG cohort: age, pre-pregnant BMI, fetal sex, gestational age, and HOMA-IR. In EPIPREG, the analysis was additionally adjusted for ethnicity. *P *< 0.05 was considered statistically significant.

### mQTL

We performed look-ups in Genetics of DNA Methylation Consortium (GoDMC) [52] of the CpGs replicated in EPIPREG to identify methylation quantitative trait loci (mQTL). The mQTLs were filtered with LD-link web-tool [53] and variants with *R*^2^ < 0.2 were kept. We used Phenoscanner [54] to search for phenotypes nominally associated (*p *< 0.05) with the mQTLs that survived filtering.

## Supplementary Information

**Additional file 1: Fig. S1.** Data distribution, at baseline and antenatal visits, based on Genomic position: open sea, shore, island, and shelf; based on gene context: TSS, exon IGR, 5'UTR, and 3'UTR.

**Additional file 2: Table S1.** Summary of number of differentially methylated probes. **Table S2.** Validation CpGs in EPIPREG cohort.

**Additional file 3: Fig. S2.** DNA methylation levels of both CpGs which responded differentially over time in the GDM group relative to the non-GDM group, at T0 and T1.

**Additional file 4: Fig. S3.** Bar plot of gene mostly enriched by significant DMP. The number in each bar indicates how many hyper- or hypo-differential methylated CpGs are included in that gene.

**Additional file 5: Fig. S4.** Receiver operating characteristic models of the best three CpGs that discriminate between GDM and non-GDM: A) Model with only one CpG cg01459453. B) Model with two CpGs: cg01459453 and cg15329406. C) Model with 3 CpGs: cg01459453, cg15329406 and cg04095097. *AUC: area under the curve*.

**Additional file 6: Fig. S5.** Receiver operating characteristic models of the three CpGs validated in EPIPREG cohort. A) One CpG model: cg04095097, B) Two CpG model: cg04095097 and cg27603605, C) Three CpG model: cg04095097, cg27603605, and cg12080079. *AUC: area under the curve*.

## Data Availability

The data sets from EPI-DG used during the current study are available from the corresponding author on reasonable request. Regarding EPIPREG due to strict regulations for genetic data and privacy protection of patients in Norway, all requests for data access are processed by the STORK G project’s steering committee. Please contact the principal investigator of STORK G (on.oiu.nisidem@dnarb.l.m.a) or the principal investigator of EPIPREG (on.oiu.nisidem@remmos.enitsirhc).
