# Supplementary figures and images for: Epigenetic marks associated with gestational diabetes mellitus across two time points during pregnancy
Source: Clin Epigenetics. 2023 Jul 6;15:110. doi: 10.1186/s13148-023-01523-8 (PMC10324212; doi:10.1186/s13148-023-01523-8)

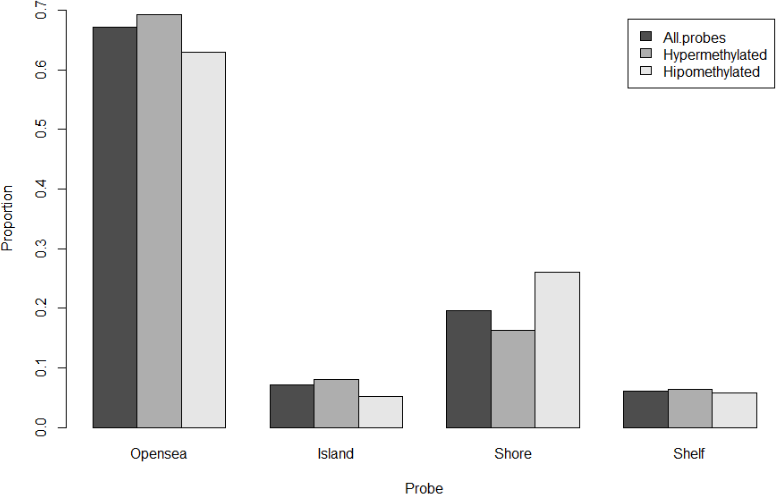

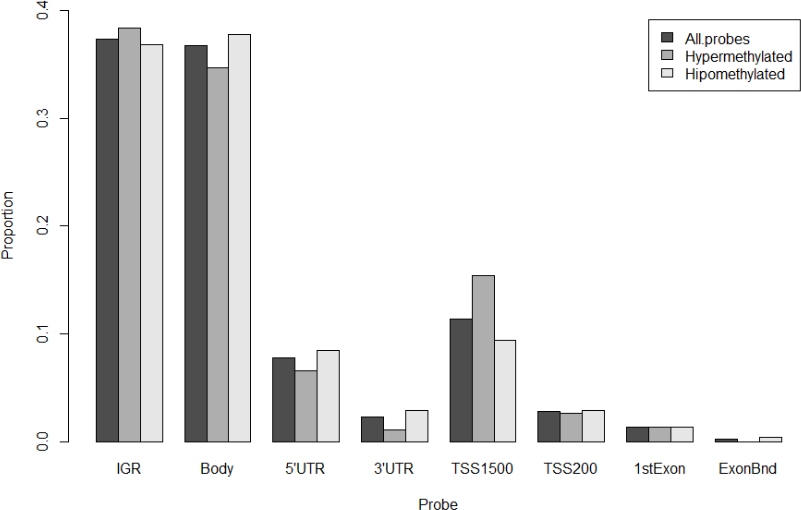
A) B)


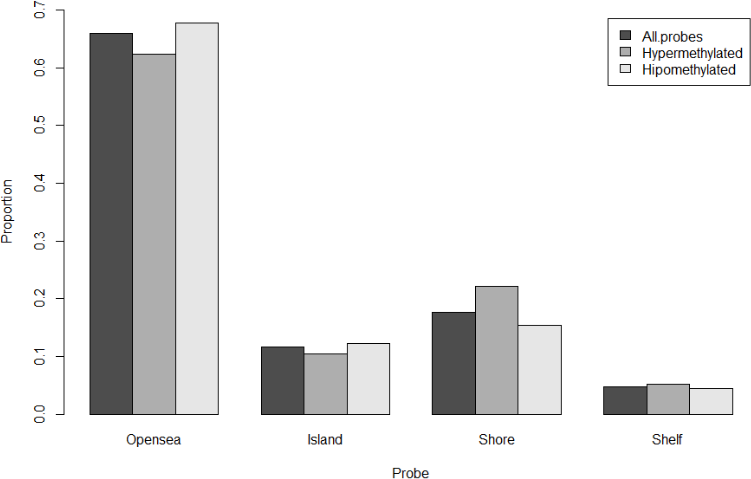

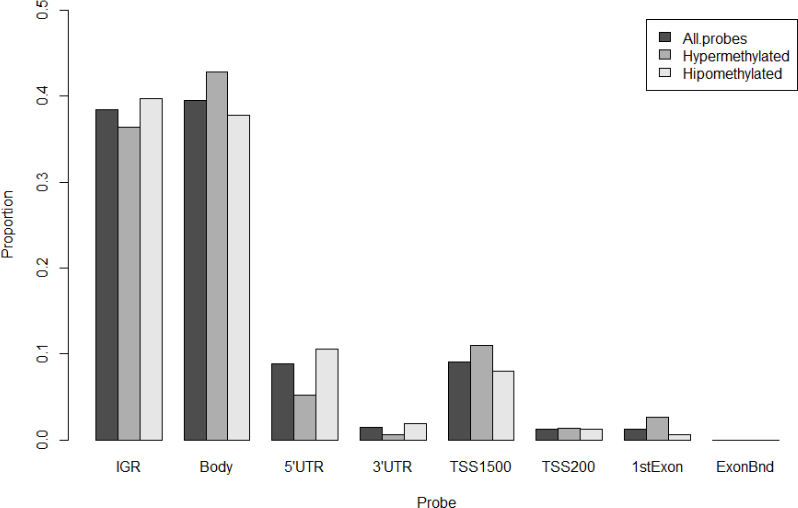
C) D)

Supplement: Supplementary file 1 — Additional file 1: Fig. S1. Data distribution, at baseline and antenatal visits, based on Genomic position: open sea, shore, island, and shelf; based on gene context: TSS, exon IGR, 5'UTR, and 3'UTR. [file 13148_2023_1523_MOESM1_ESM.docx]

A) B)


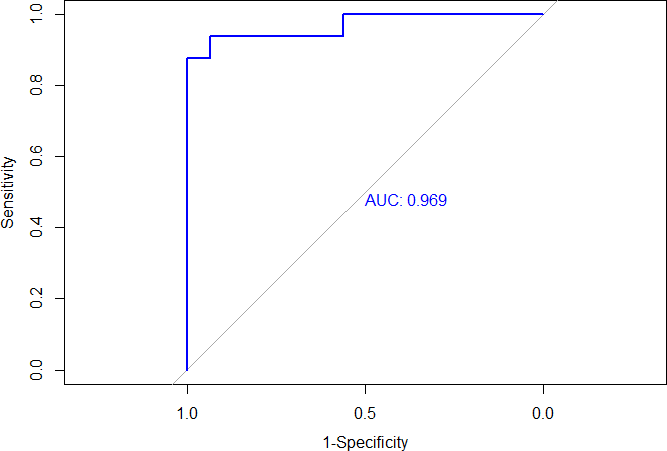

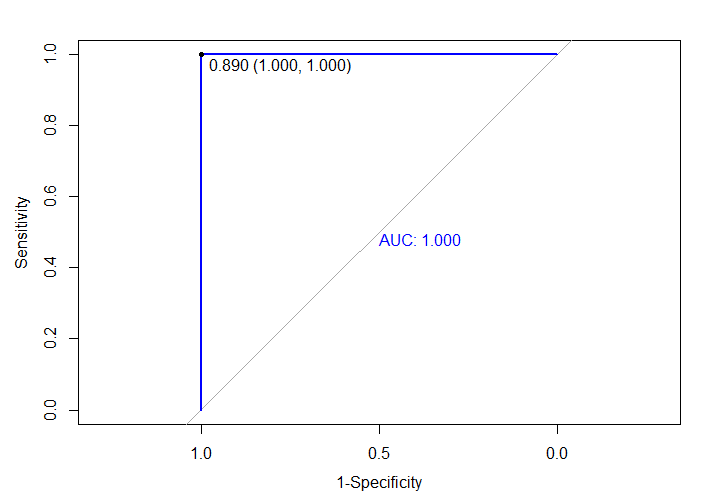

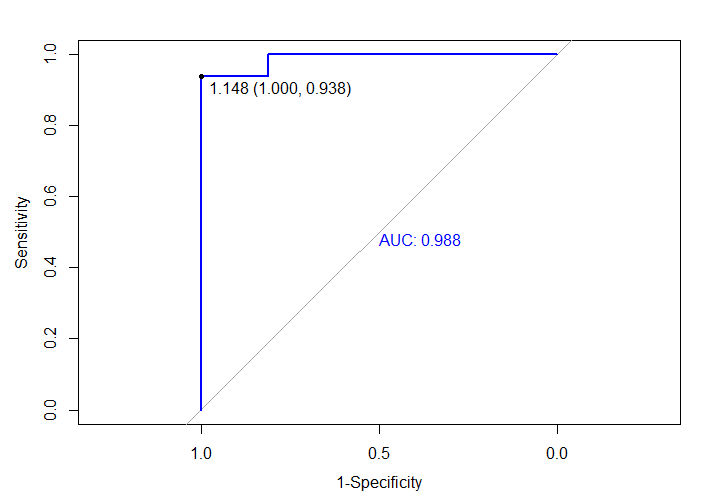


C)

Supplement: Supplementary file 5 — Additional file 5: Fig. S4. Receiver operating characteristic models of the best three CpGs that discriminate between GDM and non-GDM: A) Model with only one CpG cg01459453. B) Model with two CpGs: cg01459453 and cg15329406. C) Model with 3 CpGs: cg01459453, cg15329406 and cg04095097. AUC: area under the curve. [file 13148_2023_1523_MOESM5_ESM.docx]

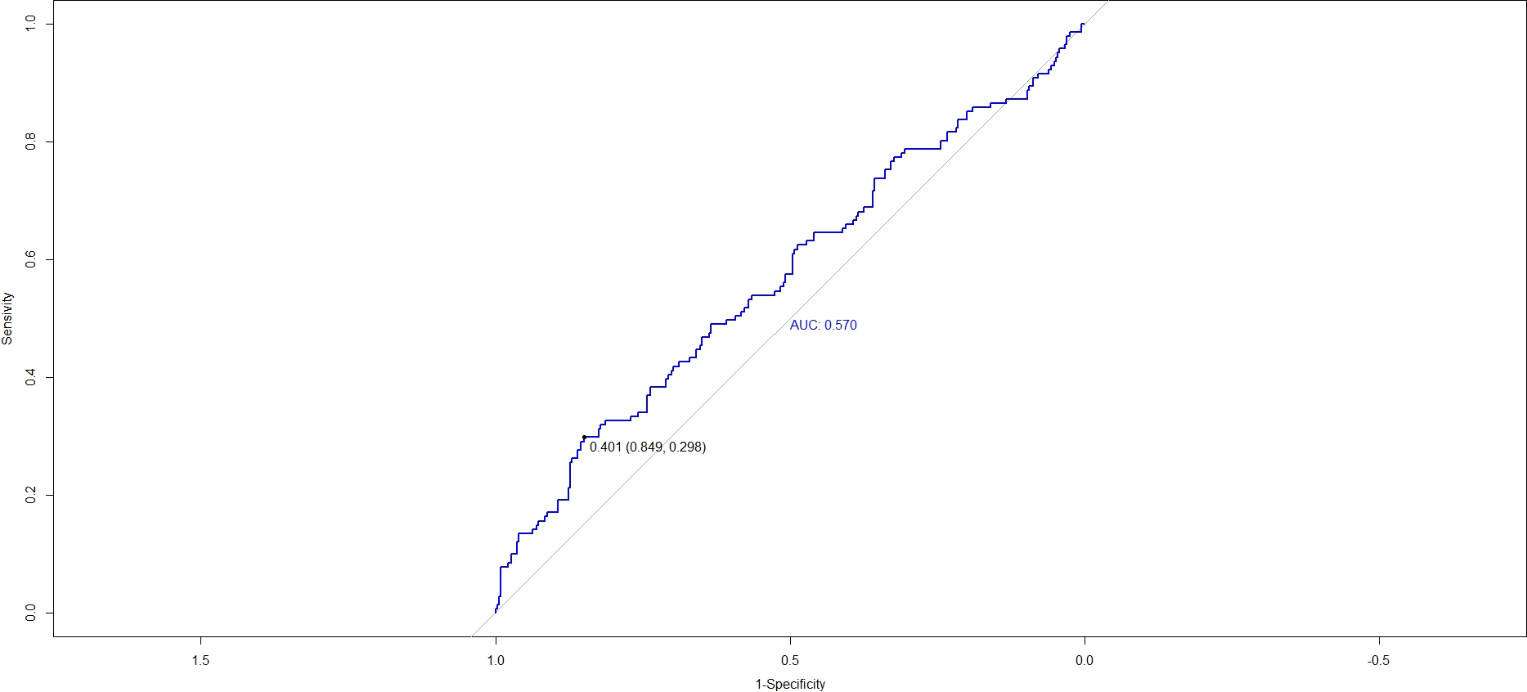

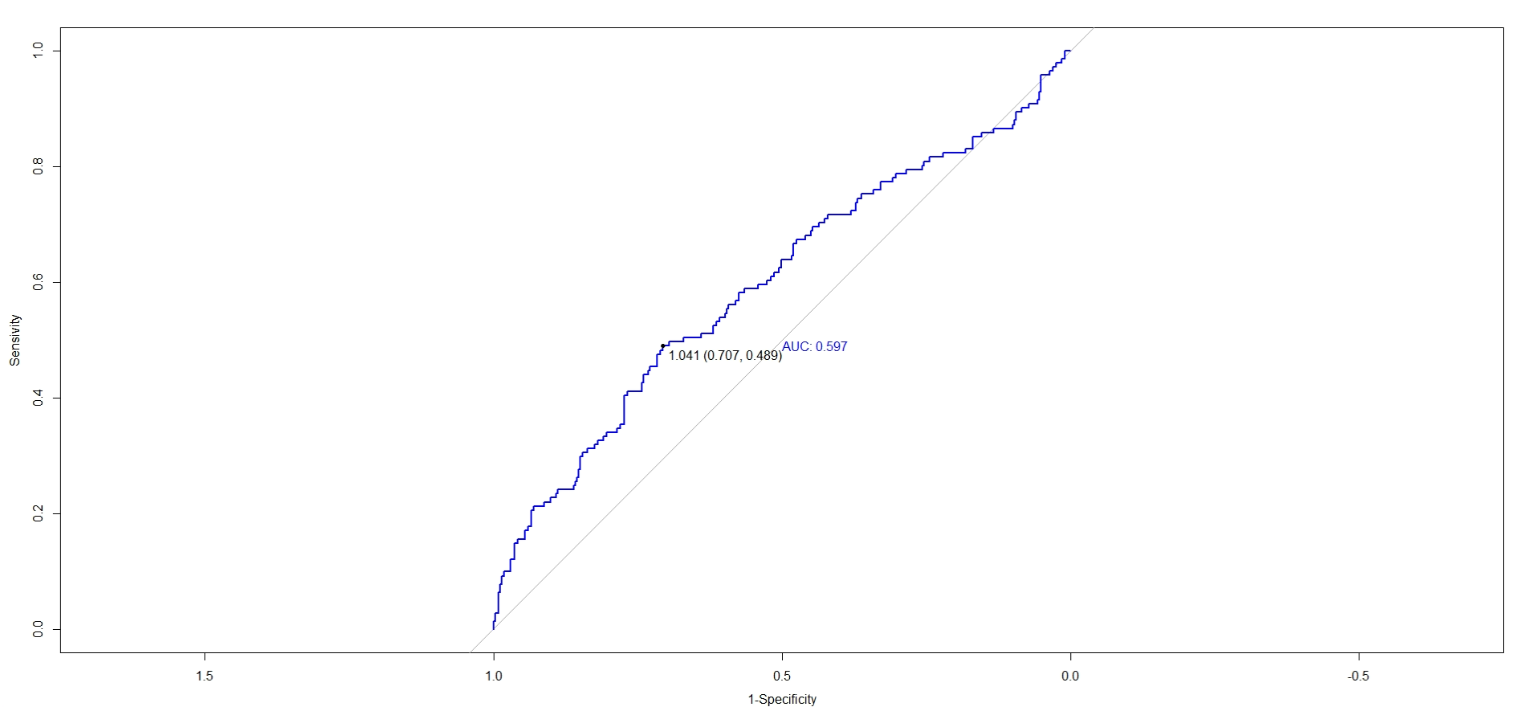


A)

B)


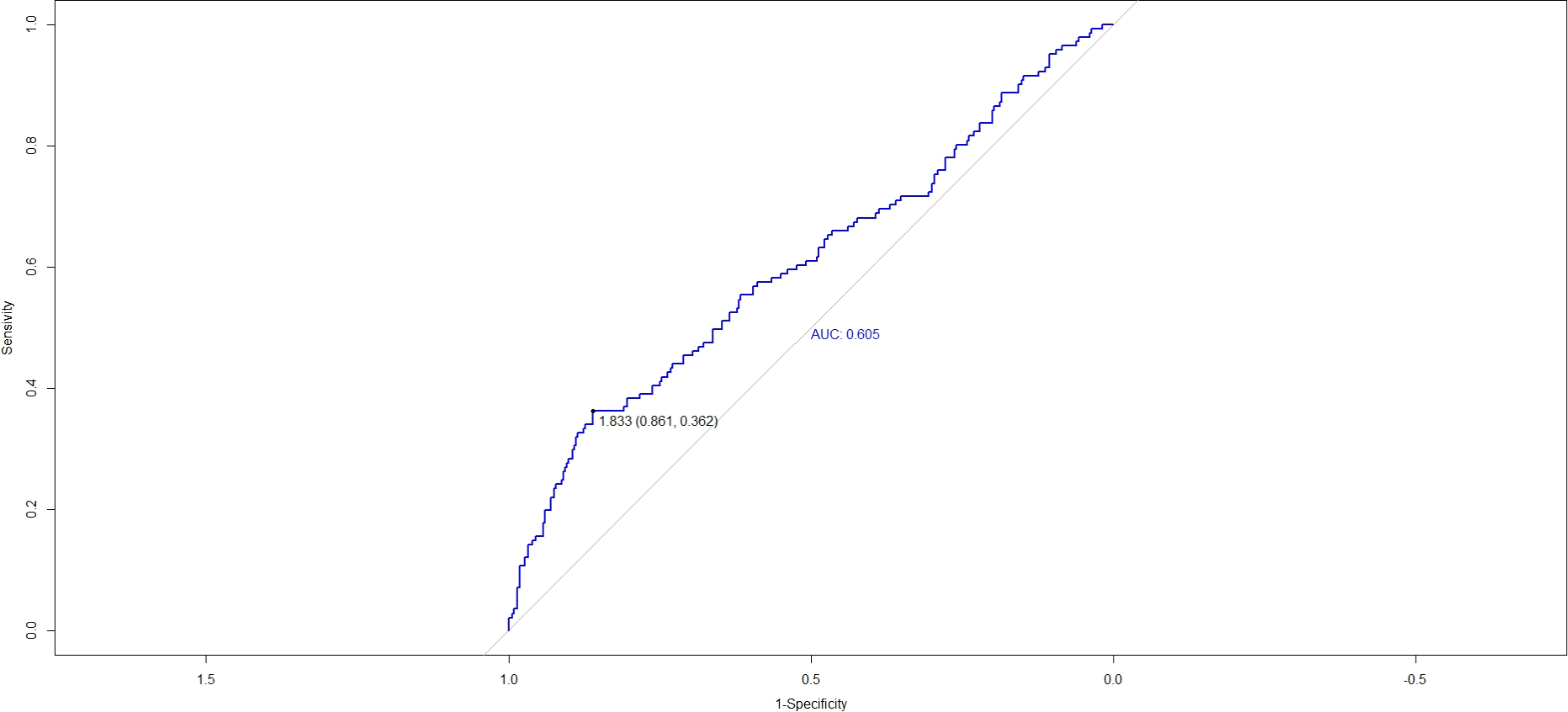
C)

Supplement: Supplementary file 6 — Additional file 6: Fig. S5. Receiver operating characteristic models of the three CpGs validated in EPIPREG cohort. A) One CpG model: cg04095097, B) Two CpG model: cg04095097 and cg27603605, C) Three CpG model: cg04095097, cg27603605, and cg12080079. AUC: area under the curve. [file 13148_2023_1523_MOESM6_ESM.docx]
