# Supplementary material for: Epigenetic marks associated with gestational diabetes mellitus across two time points during pregnancy
Source: Clin Epigenetics. 2023 Jul 6;15:110. doi: 10.1186/s13148-023-01523-8 (PMC10324212; doi:10.1186/s13148-023-01523-8)
Supplement: Supplementary file 2 — Additional file 2: Table S1. Summary of number of differentially methylated probes. Table S2. Validation CpGs in EPIPREG cohort. [file 13148_2023_1523_MOESM2_ESM.docx]

Supplementary table 1: Summary of number of Differentially Methylated Probes (DMP).

| **Comparison** | **CpG sites adjusted by age, gestational age, pre-pregnant BMI, foetal sex, weigh gain, HOMA-IR and treatment (insulin or diet**^) * ⴕ^ |
| --- | --- |
| GDM vs non-GDM at T0 | 757/384 |
| GDM vs non-GDM at T1 | 311/154 |
| T1 vs T0 in non-GDM | 263/4 |
| T1 vs T0 in GDM | 0/0 |

*False Discover Rate (FDR) p-value <0.05. ^ⴕ^Hypermethylated and Hypomethylated CpG sites

Supplementary table 2: Validation CpGs in EPIPREG cohort.

|  | EPIPREG complete | | | | EPIPREG_EU | | | | | EPIPREG_SA | | | | |
| --- | --- | --- | --- | --- | --- | --- | --- | --- | --- | --- | --- | --- | --- | --- |
| cg_ID | logFC | Mean non-GDM | Mean GDM | P.Value | cg_ID | logFC | Mean non-DGM | Mean GDM | P.Value | cg_ID | logFC | Mean Controls | Mean GDM | P.Value |
| **cg04095097** | **0.169** | **0.335** | **0.361** | **0.0042** | *cg21809624* | *-0.012* | *0.543* | *0.554* | *0.058* | **cg15589641** | **-0.025** | **0.473** | **0.463** | **0.024** |
| **cg27603605** | **0.071** | **0.669** | **0.681** | **0.016** | *cg04095097* | *0.0049* | *0.328* | *0.336* | *0.061* | **cg04095097** | **0.038** | **0.354** | **0.397** | **0.026** |
| **cg12080079** | **0.095** | **0.728** | **0.743** | **0.047** | cg08386137 | 0.0049 | 0.852 | 0.858 | 0.243 | **cg18923740** | **-0.020** | **0.308** | **0.295** | **0.040** |
| cg10102108 | 0.062 | 0.729 | 0.737 | 0.068 | cg12080079 | 0.0096 | 0.727 | 0.736 | 0.250 | cg14579430 | -0.021 | 0.462 | 0.452 | 0.075 |
| cg21809624 | 0.051 | 0.543 | 0.553 | 0.078 | cg01757548 | 0.0051 | 0.908 | 0.909 | 0.270 | cg04600077 | 0.0093 | 0.714 | 0.708 | 0.206 |
| cg01743873 | 0.049 | 0.416 | 0.425 | 0.121 | cg10102108 | 0.0054 | 0.725 | 0.73 | 0.374 | cg01757548 | 0.0058 | 0.912 | 0.909 | 0.224 |
| cg18923740 | -0.067 | 0.297 | 0.289 | 0.171 | cg14579430 | 0.008 | 0.455 | 0.444 | 0.456 | cg10102108 | 0.0078 | 0.738 | 0.747 | 0.249 |
| cg15589641 | -0.051 | 0.466 | 0.458 | 0.240 | cg12432693 | -0.0019 | 0.898 | 0.821 | 0.487 | cg07257824 | -0.022 | 0.498 | 0.512 | 0.387 |
| cg14579430 | -0.054 | 0.457 | 0.447 | 0.256 | cg04600077 | 0.0042 | 0.71 | 0.712 | 0.492 | cg01743873 | 0.0059 | 0.445 | 0.443 | 0.422 |
| cg23743013 | -0.035 | 0.362 | 0.355 | 0.283 | cg01743873 | 0.0038 | 0.404 | 0.413 | 0.557 | cg14688342 | -0.00519 | 0.674 | 0.74 | 0.442 |
| cg07257824 | -0.073 | 0.516 | 0.513 | 0.430 | cg07257824 | -0.0073 | 0.523 | 0.513 | 0.674 | cg04802986 | -0.0040 | 0.842 | 0.836 | 0.442 |
| cg04802986 | -0.024 | 0.839 | 0.837 | 0.493 | cg18923740 | 0.0034 | 0.293 | 0.285 | 0.704 | cg15329406 | 0.0058 | 0.302 | 0.294 | 0.445 |
| cg04600077 | -0.018 | 0.711 | 0.71 | 0.573 | cg27603605 | -0.0019 | 0.67 | 0.676 | 0.717 | cg18217622 | -0.015 | 0.49 | 0.513 | 0.473 |
| cg14688342 | -0.015 | 0.67 | 0.67 | 0.589 | cg23743013 | -0.0021 | 0.366 | 0.357 | 0.746 | cg01459453 | 0.0082 | 0.751 | 0.761 | 0.504 |
| cg08386137 | 0.018 | 0.854 | 0.856 | 0.593 | cg18217622 | -0.0049 | 0.514 | 0.506 | 0.748 | cg06279296 | 0.014 | 0.261 | 0.278 | 0.514 |
| cg06279296 | -0.049 | 0.253 | 0.252 | 0.633 | cg14688342 | -0.0014 | 0.668 | 0.667 | 0.804 | cg12215871 | 0.0056 | 0.811 | 0.81 | 0.545 |
| cg18217622 | -0.025 | 0.506 | 0.508 | 0.754 | cg15329406 | 0.0015 | 0.286 | 0.285 | 0.814 | cg21809624 | 0.0023 | 0.543 | 0.551 | 0.762 |
| cg15329406 | -0.0089 | 0.29 | 0.288 | 0.800 | cg06279296 | 0.0042 | 0.25 | 0.234 | 0.824 | cg12432693 | -0.00099 | 0.9 | 0.899 | 0.768 |
| cg01459453 | -0.014 | 0.751 | 0.752 | 0.810 | cg01459453 | 0.0015 | 0.752 | 0.746 | 0.873 | cg27603605 | 0.0019 | 0.667 | 0.688 | 0.801 |
| cg12215871 | 0.011 | 0.817 | 0.816 | 0.819 | cg04802986 | -220.00 | 0.838 | 0.836 | 0.885 | cg12080079 | -0.00087 | 0.728 | 0.753 | 0.928 |
| cg01757548 | 0.0079 | 0.909 | 0.909 | 0.863 | cg12215871 | 0.00082 | 0.82 | 0.821 | 0.899 | cg23743013 | -0.00073 | 0.354 | 0.355 | 0.931 |
| cg12432693 | 0.00026 | 0.899 | 0.898 | 0.993 | cg15589641 | 0.00077 | 0.463 | 0.456 | 0.936 | cg08386137 | -0.00025 | 0.858 | 0.853 | 0.960 |

logFC: log_2_ Fold Change. EPIPREG completed: Analysis in all the cohort. EPIPREG_EU: Analysis only in European cohort. EPIPREG_SA: Analysis only in South Asians cohort
