# Supplementary material for: Epigenetic marks associated with gestational diabetes mellitus across two time points during pregnancy
Source: Clin Epigenetics. 2023 Jul 6;15:110. doi: 10.1186/s13148-023-01523-8 (PMC10324212; doi:10.1186/s13148-023-01523-8)
Supplement: Supplementary file 4 — Additional file 4: Fig. S3. Bar plot of gene mostly enriched by significant DMP. The number in each bar indicates how many hyper- or hypo-differential methylated CpGs are included in that gene. [file 13148_2023_1523_MOESM4_ESM.docx]

B)

A)
