# Supplementary material for: Epigenetic marks associated with gestational diabetes mellitus across two time points during pregnancy
Source: Clin Epigenetics. 2023 Jul 6;15:110. doi: 10.1186/s13148-023-01523-8 (PMC10324212; doi:10.1186/s13148-023-01523-8)
Supplement: Supplementary file 4 [file 13148_2023_1523_MOESM3_ESM.docx]

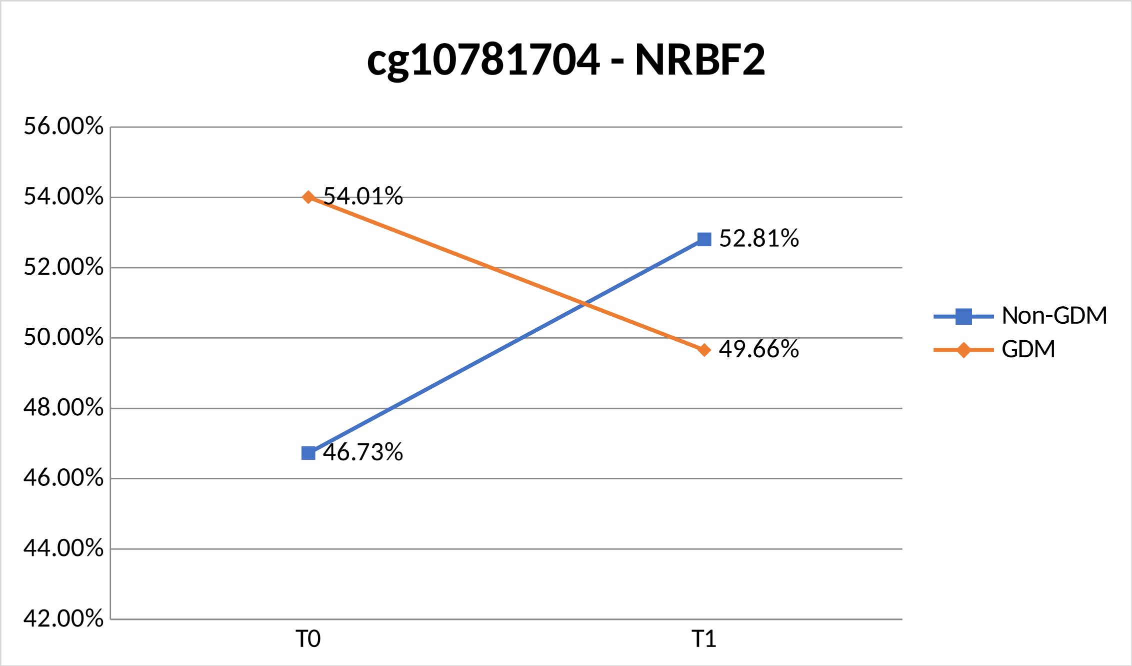
cg10781704 - NRBF2
56.00%
54.00%
52.00%
50.00%
48.00%
46.00%
44.00%
42.00%
54.01%
52.81%
49.66%
46.73%
T0
T1
Non-GDM
GDM

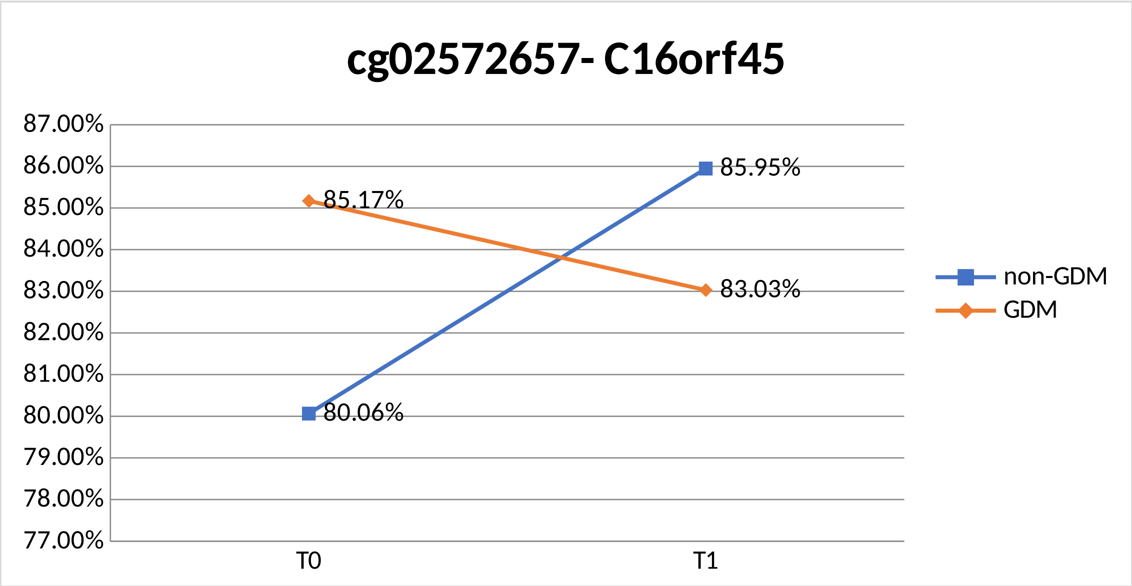
cg02572657- C16orf45
87.00%
86.00%
85.00%
84.00%
83.00%
82.00%
81.00%
80.00%
79.00%
78.00%
77.00%
85.95%
85.17%
83.03%
80.06%
T0
T1
non-GDM
GDM
